# Advances in Immunotherapy and the TGF-β Resistance Pathway in Metastatic Bladder Cancer

**DOI:** 10.3390/cancers13225724

**Published:** 2021-11-16

**Authors:** David J. Benjamin, Yung Lyou

**Affiliations:** 1 Chao Family Comprehensive Cancer Center, Division of Hematology/Oncology, Department of Medicine, UC Irvine Medical Center, Orange, CA 92868, USA; djbenjam@hs.uci.edu; 2 Department of Medical Oncology and Experimental Therapeutics, City of Hope Comprehensive Cancer Center, Duarte, CA 91010, USA

**Keywords:** urothelial carcinoma, bladder cancer, immunotherapy, TGF-β resistance pathway

## Abstract

**Simple Summary:**

Bladder cancer accounts for a significant burden to global public health. Despite advances in therapeutics with the advent of immunotherapy, only a small subset of patients benefit from immunotherapy. In this review, we examine the evidence that suggests that the TGF-β pathway may present a resistance mechanism to immunotherapy. In addition, we present possible therapies that may overcome the TGF-β resistance pathway in the treatment of bladder cancer.

**Abstract:**

Bladder cancer accounts for nearly 200,000 deaths worldwide yearly. Urothelial carcinoma (UC) accounts for nearly 90% of cases of bladder cancer. Cisplatin-based chemotherapy has remained the mainstay of treatment in the first-line setting for locally advanced or metastatic UC. More recently, the treatment paradigm in the second-line setting was drastically altered with the approval of several immune checkpoint inhibitors (ICIs). Given that only a small subset of patients respond to ICI, further studies have been undertaken to understand potential resistance mechanisms to ICI. One potential resistance mechanism that has been identified in the setting of metastatic UC is the TGF-β signaling pathway. Several pre-clinical and ongoing clinical trials in multiple advanced tumor types have evaluated several therapies that target the TGF-β pathway. In addition, there are ongoing and planned clinical trials combining TGF-β inhibition with ICI, which may provide a promising therapeutic approach for patients with advanced and metastatic UC.

## 1. Introduction

Urothelial cancer (UC) is a significant public health burden with approximately 550,000 newly diagnosed patients and accounts for nearly 200,000 deaths globally each year [1,2]. This is an aggressive disease, where 25% of patients who receive localized disease treatment will unfortunately later progress to develop recurrent metastatic disease [3]. Cytotoxic platinum-based chemotherapy regimens followed by switch maintenance to the immune checkpoint inhibitor (ICI) avelumab is the preferred first-line choice of treatment for metastatic UC (mUC) [4,5,6]. In addition, for those patients who have been found to be ineligible or progressed on first-line platinum-based chemotherapy regimens, second-line treatment with the use of immune checkpoint inhibitors (ICIs) has also demonstrated superior clinical outcomes compared to second-line chemotherapies such as paclitaxel [7,8,9]. As a result, current NCCN guidelines (version 3.2021) have assigned pembrolizumab a category 1 recommendation in the second-line setting for mUC treatments. Both atezolizumab and durvalumab were voluntarily withdrawn for the indication of second-line therapy for mUC by Roche and AstraZeneca respectively, in early 2021, and are currently undergoing re-evaluation for continual FDA approval [10,11]. Among patients with mUC treated with an ICI, only about 20% of patients respond to treatment [12]. This means that the majority of patients (~80%) do not respond to ICIs and will need further treatment with other later line agents. This lack of response to ICIs has been observed in many other cancers [13]. Consequently, this has led to considerable interest in identifying ICI resistance pathways.

There are several proposed ICI resistance pathways, including TGF-β, PTEN, MYC, WNT, VEGF, and FGF [13,14,15]. Of these pathways, the TGF-β signaling pathway has been found to be a potential driver of ICI resistance, specifically in the context of metastatic UC [16]. In this review, we will (i) discuss the pathophysiology of the TGF-β signaling pathway with regards to cancer progression and evidence of ICI resistance, (ii) discuss the pre-clinical and clinical evidence supporting the combination of ICI with TGF-β inhibitors to overcome resistance, and (iii) examine current ongoing clinical trials using this combination treatment and propose future directions.

## 2. TGF-B Signaling and Cancer Progression

### 2.1. TGF-β Signaling

**Figure 1:**
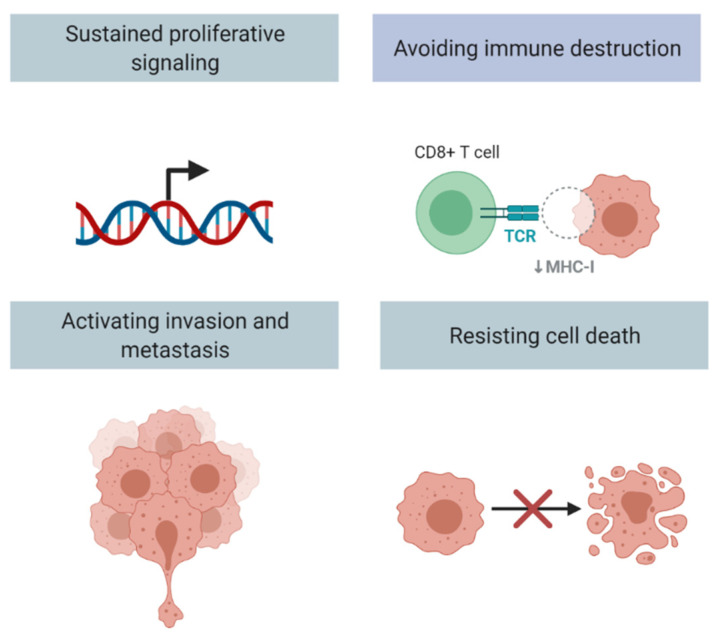
TGF-β pathway and related hallmarks of cancer (adapted from “Hallmarks of Cancer”, by BioRender.com (2021). Retrieved from https://app.biorender.com/biorender-templates, accessed on 14 October 2021).

**Figure 2:**
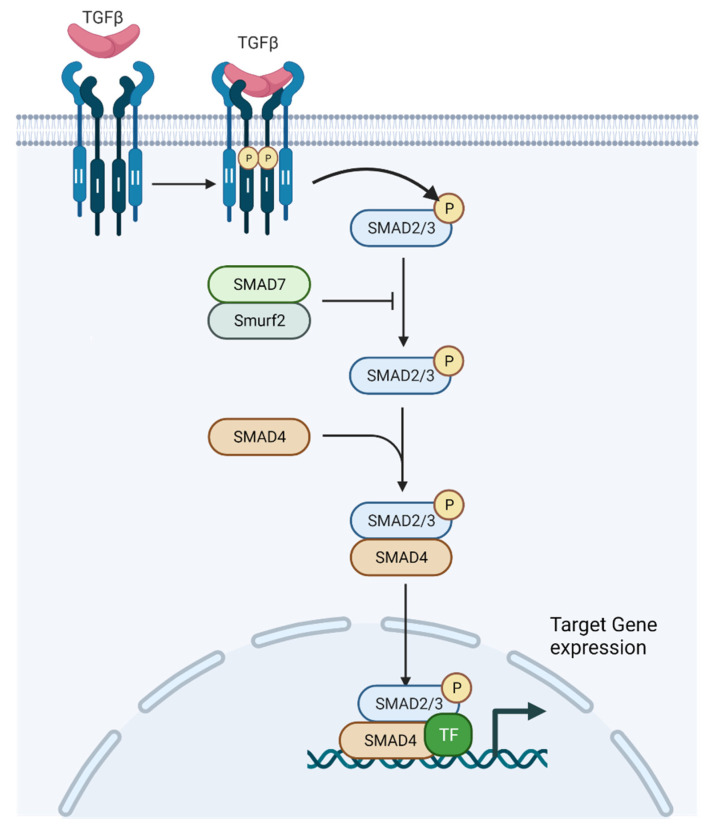
TGF-β signaling pathway (adapted from “TGF-β signaling”, by BioRender.com (2021). Retrieved from https://app.biorender.com/biorender-templates, accessed on 14 October 2021).

The transforming growth factor-β (TGF-β) signaling pathway is a highly conserved and complex pathway which has been found to be involved in several hallmarks of cancer, such as resisting cell death, evading growth suppressors, inducing angiogenesis, and activating invasion and metastasis (Figure 1) [17,18,19,20,21,22,23,24]. The components of the signaling pathway have been described in more depth in other recent reviews, and are only briefly discussed here as it is not the intended focus of this manuscript [25,26]. Since its initial discovery, there has been significant interest in understanding the TGF-β pathway given its pleotropic roles in the development and progression of cancer. There have been several observations of the TGF-β pathway in all solid tumor types, including: TGF-β expression is increased in tumor cells, and TGF-β signaling promotes cancer cell invasion and dissemination [27]. The mammalian genome encodes three TGF-β proteins: TGF-β1, TGF-β2, and TGF-β3. TGF-β1 is notably enriched in tumor cells, myeloid-derived suppressor cells (MDSCs), and carcinoma-associated fibroblasts (CAFs) [16]. This is of particular interest as the presence of MDSCs and CAFs have also been associated with tumor progression. Latent complex of TGF-β can be deposited as inactive forms in the extracellular matrix (ECM) when covalently linked to a fibrillin-like latent TGF-β binding protein 1 (LTBP1) [28]. Several integrins subsequently play a role in activating TGF-β1, including avβ6 and avβ1 at the surface of epithelial cells and fibroblasts respectively, and possibly avβ8 in or near cancerous cells [29,30,31]. After activation, TGF-β binds a tetrameric combination of two types of transmembrane kinases, type I and II receptors (TβRI and TβRII) (Figure 2). TGF-β activates these receptor complexes and ultimately Smad2 and Smad3 through phosphorylation by TβRI (Figure 2) [32]. Consequently, these Smads, in addition to Smad4, translocate into the nucleus and combine with transcription factors and co-regulators in order to activate or repress target gene transcription [33,34]. In addition to its roles in gene transcription, TGF-β affects microRNA expression as well as Smad-mediated control of microRNA maturation [35,36]. These microRNAs, in turn, affect the translation of many target gene transcripts, including gene pathways linked to cancer progression such as Myc, PI3K/Akt, Notch, and Wnt [37]. Specifically, TGF-β1 has been demonstrated to stimulate expression of ZEB1-AS1, which leads to a decrease in expression of miR-200b, which is a tumor suppressor [38]. ZEB1-AS1, in turn, promotes the cell cycle and leads to the inhibition of apoptosis. In addition, fascin1, which is a target of miR-200b, leads to activated migration and invasion in bladder cancer cells. Furthermore, TGF-β1 can lead to the expression of miR-221 that in turn leads to EMT, and thus contributes to the invasiveness of bladder cancer cells [39]. Of note, TGF-mRNA as well as TGF-β1 protein and its receptor (TGF-βR1) were overexpressed in urine samples of patients with bladder cancer compared to healthy control individuals [40].

### 2.2. TGF-β Pathway and Fibroblasts

CAFs promote tumorigenesis through the secretion of cytokines and chemokines, as well as through effects on the TME [41]. Recent research has demonstrated that CAFs are heterogenous and that there are several subtypes of CAFs based on tumor origin [42]. The molecular signatures of these subtypes have been shown to vary, and may have implications in prognosis and treatment [43]. CAFs play a crucial role in the TME through ECM remodeling, secretion of cytokines, and several other activities. In non-cancerous tissue, fibroblasts exhibit low levels of proliferation; however, in response to inflammation or injury, fibroblasts can be “activated” and demonstrate proliferation and activity [44,45]. Similarly, CAFs demonstrate activity that is representative of activated fibroblasts. In turn, stromal fibroblasts play a key role in the architecture of the TME through secretion of ECM proteins and remodeling of the ECM [27]. It has also been noted that stromal fibroblasts are a source of TGF-β, with TGF-β signaling inducing changes in their physiology [46]. Of note, TGF-β at low concentrations acts as a chemoattractant that recruits more fibroblasts [47,48]. The activated TGF-β signaling in fibroblasts can also promote cell survival and protect against cell death (Figure 1), playing a role in cancer progression [49,50].

### 2.3. TGF-β Pathway Can Drive Cancer Metastases and Drug Resistance via Epithelial Mesenchymal Transformation (EMT)

Epithelial mesenchymal transformation (EMT), the process by which epithelial cells acquire mesenchymal features, increases tumor invasiveness and metastatic activity, ultimately driving cancer progression (Figure 1) [51]. TGF-β signaling promotes epithelial cells to transform to the mesenchymal phenotype. These EMT changes promote migration and invasion through the ECM [27]. TGF-β promotes EMT through TβRI activation, and in turn, Smad3/4-mediated transcription of genes for transcription factors such as Snail1, Snail2, ZEB1, and ZEB2, which activate mesenchymal genes and repress epithelial cell genes [52]. EMT is also dependent on several pathways, including WNT and MAPK, in addition to MTOR signaling downstream of AKT [52,53]. While full EMT changes in cancer are rare, cancers may have a partial phenotype consisting of both epithelial and mesenchymal cells.

Several studies have specifically evaluated how TGF-β signaling promotes EMT in bladder cancer. TGFBI, an exocrine protein that has been linked to the development of multiple tumor types including pancreatic cancer, was found to be elevated in muscle-invasive bladder cancer (MIBC) compared to non-MIBC, and can lead to EMT in vitro through upregulation of EMT genes, including Snail, Slug, Vimentin, MMP2, and MMP9 genes [54]. In addition to TGFBI, the actin-binding protein transgelin appears to play a role in EMT through the TGF-β signaling pathway. A recent study found that transgelin is highly expressed in bladder cancer, and promotes EMT both in vivo and in vitro [55]. Furthermore, the study also found that there was a significant correlation between the EMT transcription factor Slug with transgelin in bladder cancer. As such, there are several genes and proteins that play a role in EMT in bladder cancer through TGF-β signaling.

EMT has been shown to increase the number of cells with stem cell properties, also known as cancer stem cells (CSCs) [56]. CSCs are significant as some are inherently resistant to traditional treatments such as chemotherapy, as well as to newer treatments such as ICI through the secretion of regulatory factors [57,58]. These cells are able to initiate tumor formation in vivo. In addition to its role in increasing CSCs, EMT exerts local immunosuppression in the tumor microenvironment (TME) through several mechanisms [59,60]. First, EMT causes repression of MHC class I-mediated antigen presentation by tumor cells, and therefore indirectly suppresses the cytotoxic activity of CD8+ T cells due to their lack of recognition of tumor cells [59,60]. In addition, EMT leads to increased expression of immunosuppressive chemokines and cytokines such as TGF-β1, as TGF-β promotes Treg cell differentiation [59,60].

In 2016, Liang and colleagues studied the TGF-β signaling pathway using an induced murine bladder cancer model [61]. The researchers used the mouse model of KRT4-Cre-driven conditional knockout of TGF-β2, and induced bladder cancer using N-butyl-N-4-hydroxylbutyl nitrosamine (BBN). While all of the control mice developed MIBC, it developed in only 37.5% of the conditional knockout mice. They calculated the relative gross bladder/body weight ratio to estimate the tumor growth in the murine model, and found that the average ratio in the control group was 4.65% ± 1.31% compared to 2.23% ± 1.00% (*p* = 0.022). In addition, they found that K14-expressing tumor cells, which were previously identified as cancer stem cells, were found to be decreased in the conditional knockout tumors in comparison to the control murine model. In addition, researchers found that TGF-βR2 conditional knockout mice had a reduction in EMT marker genes such as Vimentin, Slug, Snai1, Twist, and Zeb1, thus underscoring the role of TGF-β signaling on EMT. Finally, researchers used a small-molecule LY364947, an inhibitor of TGBR-1, at a dose of 1 mg/kg three times a week for 4 weeks, injected intraperitoneally. The number of proliferating cells, as defined by Ki-67+ cells, were decreased in tumors treated with LY364947.

In addition to EMT leading to increased resistance to chemotherapy, EMT has also been correlated with resistance to ICI [62,63,64]. EMT increases the expression of PD-L1 on cancer cells and thereby inhibits CD8+ T lymphocyte activation [65,66]. EMT and resistance to PD-L1 blockade has been specifically studied in the setting of metastatic urothelial carcinoma treated with the ICI nivolumab [67]. Wang and colleagues found that in urothelial cancer tumors with T cell infiltration, high EMT gene expression was associated with shorter progression-free survival (PFS) and overall survival (OS), as well as lower response rates in patients with mUC receiving ICI. Thus, it can be surmised that the TGF-β signaling pathway which promotes EMT plays a role in resistance to ICI.

## 3. TGF-β Signaling Affects the Tumor Immune Microenvironment

The tumor immune microenvironment has been found to be correlated with ICI treatment response. “Hot” tumors, which are also known as immune-inflamed phenotype, are defined by T cells present in the tumor center and more responsiveness to treatment with ICI. On the contrary, “cold” tumors, which are also known as immune-desert phenotype, are defined by the absence of T cells, a lack of T cell priming, and defective antigen presentation. These tumors generally have a low response to ICI. Immune-excluded phenotype tumors are defined by the presence of T cells in the tumor stroma, and have a response to ICI in between tumor-inflamed and tumor-desert phenotypes [68]. The TGF-β signaling pathway has been shown to exert immunosuppressive effects in the TME. TGF-β signaling affects several cell types, including macrophages, neutrophils, and NK cells. TGF-β1 represses NKG2D expression, which plays a role in inducing activation and cytotoxic potential in NK cells [69,70,71,72]. TGF-β signaling also decreases the expression of IFN-γ and TBET in NK cells and thereby dampens Th1 responses [73]. In addition, TGF-β-Smad signaling represses INF-γ, the cytokine that aids in CD8+ T cell proliferation [74]. Moreover, TGF-β also represses antigen presentation and promotes immunosuppressive activity in dendritic cells (DCs) [75,76,77]. TGF-β also may inhibit the inflammatory response of the NF-KB through degradation of MYD88 [78]. Furthermore, research also indicates that the TGF-β pathway may also inhibit the activity of inflammatory phase macrophages through the TNF pathway and Smad7 [79]. Finally, TGF-β also suppresses the production of reactive oxygen species (ROS), which is critical for the activity of M1 macrophages present in tumors [80,81].

As previously discussed, TGF-β has direct and indirect effects on CD8+ T cell-mediated immunity. The TGF-β signaling pathway suppresses several processes that lead to CD8+ T cell activation, including antigen processing and presentation, such as MHC and HLA in antigen-presenting cells (APCs) [75,82,83]. In addition, TGF-β also suppresses IL-2 expression, which is needed for CD8+ T cell proliferation [84]. TGF-β also inhibits CD8+ T cell activity by affecting lytic function through downregulation of perforin, granzymes A and B, and INF-γ [85]. Finally, as the role of CD4+ T cell lymphocytes in immune surveillance and activity against cancerous cells becomes more apparent, with recent evidence suggesting a role in advancing and maintaining anti-cancer activity, TGF-β appears to suppress CD4+ T_H_ cell proliferation [86,87]. As such, it is evident that the TGF-β signaling pathway plays several roles in immunosuppression on a systemic level and in the TME.

## 4. TGF-β Pathway Induced Immune Cell Exclusion and Immune Checkpoint Inhibitor Resistance

The TGF-β signaling pathway has been found to be correlated with resistance to ICI [88]. The IMvigor210 study was a multi-center, single-arm phase II clinical trial that evaluated the anti-PD-L1 inhibitor, atezolizumab, in the first-line setting in treating patients with locally advanced or metastatic UC who were ineligible for cisplatin-based chemotherapy treatment [89]. The study investigators evaluated pre-treatment tumor samples to identify biomarkers of treatment response [16]. Patients with a partial or complete response were treated as responders, whereas patients who had stable or progressive disease were considered to be non-responders. This study found that high tumor mutational burden (TMB) and the presence of CD8 + effector T cells were correlated with PD-L1 response. Of clinical relevance, this gene set was associated with response and survival outcomes, including overall survival (OS). The investigators also found that the genes associated with the TGF-β signaling pathway were strongly associated with ICI non-response. In fact, two key TGF-β pathway genes, *TGFB1*, a TGF-β ligand, and *TGFBR2*, a TGF-β receptor, were found to have increased expression in tumor samples of non-responders, and to be associated with reduced OS.

**Figure 3:**
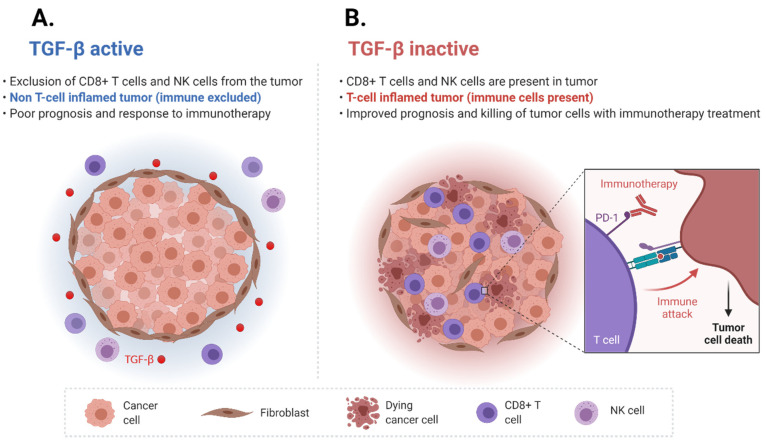
TGF-β signaling pathway drives immune cell exclusion. (**A**) Active TGF-β signaling pathway drives immune cell exclusion. (**B**) Inactive TGF-β signaling pathway drives a T cell-inflamed tumor phenotype (adapted from “Cold vs. Hot tumors”, by BioRender.com (2021). Retrieved from https://app.biorender.com/biorender-templates, accessed on 14 October 2021).

The investigators further studied the patients’ tumor tissue biopsies to identify tumor-immune phenotypes, including immune desert, immune-excluded, and immune-inflamed phenotypes. They found that in immune-excluded tumors, CD8+ T cells were in close proximity to the desmoplastic stroma. They measured the activity of the TGF-β pathway in fibroblasts using a pan-fibroblast TGF-β response signature (F-TBRS) and found that expression of this signature was higher in immune-excluded tumors. As such, the TGF-β pathway was demonstrated to be associated with exclusion of CD8+ T cells from the tumor tissue itself and instead were found outside in the surrounding fibroblast-rich and desmoplastic stroma. These findings suggested that the TGF- β pathway could drive immune cell exclusion and ICI non-response (Figure 3).

To directly investigate the hypothesis that the TGF-β pathway could drive immune cell exclusion and ICI non-response, the investigators then directly evaluated the effects of TGF-β signaling using the EMT6 murine model. The EMT6 murine model is a triple-negative breast cancer cell line that is known to be non-responsive to ICI as well as have immune cell exclusion in tumors [90]. The investigators used a custom murine anti-PD-L1 and anti-TGF-β antibody to test this hypothesis. The authors reported that combining TGF-β blockade with ICI led to a significant increase in tumor-infiltrating T cells, such as CD8+ effector cells. Moreover, the distribution of T cells changed with the mean distance from the tumor center decreasing, whereas the mean distance from the stromal border increased. Finally, dual blockade of TGF-β and PD-L1 led to a reduction in F-TBRS score and expression of fibroblast genes that are associated with matrix remodeling. Most impressively, the dual blockade led to tumor growth regression. These findings were also reproduced in a second murine model, MC38, which showed a 70% complete response (CR) rate in mice treated with anti-PD-L1 and anti-TGF-β therapies as opposed to a CR rate of 0% with anti-PD-L1 therapy alone. Tumor responses to treatment were measured through several methods, including by measurement with calipers two times a week for eight weeks total, as well as tumor weights at seven days after treatment. As such, ICI in combination with inhibition of the TGF-β pathway led to T cell infiltration and reprogramming of stromal fibroblasts, suggesting that it could convert an immune-excluded into an immune-inflamed tumor phenotype. This study has provided the scientific rationale to pursue clinical studies employing inhibition of the TGF-β pathway as a way to potentiate ICI treatment response for patients with mUC.

## 5. Overcoming ICI Resistance through the Use of TGF-β Inhibition

**Figure 4:**
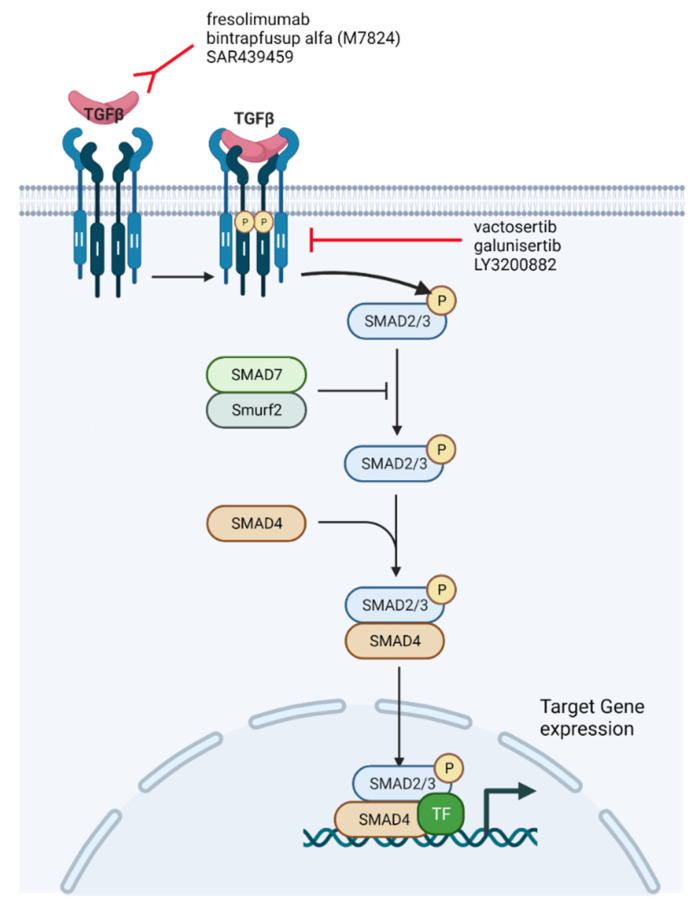
Current TGF-β signaling inhibitors being used in combination with ICI for human clinical trials (adapted from “TGF-β signaling”, by BioRender.com (2021). Retrieved from https://app.biorender.com/biorender-templates, accessed on 14 October 2021).

Given the significant amount of evidence surrounding the role of the TGF-β pathway in cancer progression and as a mechanism of resistance to ICI, there has been considerable interest in the development of therapeutics that inhibit the TGF-β pathway. There are several major classes of TGF-β pathway inhibitors, including: antibodies that prevent TGF-β binding to receptors, molecules that inhibit TGF-β receptor kinases, TGF-β ligand traps, and other therapies that interfere with latent TGF-β pathway complexes (Figure 4) [27]. Several pre-clinical and phase I clinical trials have been conducted with these therapies, with promising results that have led to ongoing phase II clinical trials. There are currently several small-molecule inhibitors and monoclonal antibodies targeting the TGF-β pathway. Most of these agents were first being studied as monotherapies, and are now being studied in the setting of combination therapy with ICIs for several tumor types, including UC.

One particular treatment type being used to act on the TGF-β pathway are monoclonal antibodies. Monoclonal antibodies such as fresolimumab prevent TGF-β binding to receptors. Fresolimumab is a human anti-TGF-β IgG4k monoclonal antibody that acts on all three TGF-β isoforms, including TGF-β1, TGF-β2, and TGF-β3 [91]. The antibody was studied in the phase I setting in patients with metastatic melanoma and metastatic renal cell carcinoma, and found to have no dose-limiting toxicity (DLT) at 15 mg/kg. Fresolimumab was administered intravenously once every 4 weeks. Of 29 patients in the trial, 1 patient had a partial response while 6 patients had stable disease with a median PFS of 24 weeks [91]. Observed adverse events include bleeding (4 patients), epistaxis (4 patients), headaches (4 patients), and fatigue (3 patients). SAR439459 is a next-generation antibody which acts on all three TGF-β isoforms. In vitro studies combining SAR439459 with anti-PD-L1 have demonstrated tumor regression and improved CD8+ T cell proliferation and response [92]. NIS973 is an anti-TGF-β antibody that selectively acts on TGF-β1 and TGF-β2, but not on TGF-β3 given the possibility that TGF-β3 opposes the effects of TGF-β1 and TGF-β2 [93]. This antibody is being further tested in a phase I/Ib clinical trial in combination with the anti-PD-1 antibody spartalizumab. Several completed clinical trials involving antibodies that prevent TGF-β binding to receptors are detailed further in Table 1, and ongoing clinical trials are detailed in Table 2.

An additional treatment type being used in acting on the TGF-β pathway is small-molecular inhibitors. Galunisertib and vactosertinib are both small-molecular receptor kinase inhibitors, with LY3200882 being a second-generation derivative of galunisertib. These inhibitors prevent the binding of ATP to TGF-β receptors, consequently blocking Smad2 and Smad3 activation in response to TGF-β [94,95]. Although these small molecules act on Smad activation and inhibit the downstream effects of TGF-β signaling, it is unclear what role they play in TGF-β-induced PI3K-AKT-mTOR activation. Both inhibitors are currently being investigated in early-phase clinical trials (Table 1). Galunisertib has been studied in several malignancies, including glioma and hepatocellular carcinoma (HCC) [96,97]. The single-agent galunisertib, administered orally twice a day on a 14 days on/14 days off cycle, was found to be associated with clinical response (complete response + partial response + stable disease) in 12/56 (21.4%) patients with glioma [98]. Additionally, galunisertib has been studied in combination with sorafenib in the setting of unresectable HCC. In a phase Ib study conducted in Japan, Ikea and colleagues found 11 patients with stable disease and 1 with a partial response [99]. The most common grade 3 or higher treatment-related adverse events were hypophosphatemia (10 patients, 71.4%) and hand-foot syndrome (7 patients, 50%). Galunisertib has been studied in the setting of multiple advanced tumors in combination with ICI, including: in combination with durvalumab for metastatic pancreatic cancer (NCT02734160), as well as in combination with nivolumab for advanced treatment refractory solid tumors, and recurrent/refractory NSCLC or HCC (NCT02423343). Additionally, galunisertib is being studied in combination with chemotherapy or androgen blockade in several studies: in combination with paclitaxel for metastatic triple-negative breast cancer (NCT02672475) and in combination with metastatic castrate-resistant prostate cancer (NCT02452008). There are also several ongoing phase I/II clinical trials involving vactosertib as monotherapy or in combination, including: combination with pembrolizumab in PD-L1-positive non-small-cell lung cancer (NCT04515979), in combination with durvalumab in urothelial carcinoma (NCT04064190), and in combination with pembrolizumab for metastatic colorectal or gastric cancer (NCT03724851). As such, small-molecule inhibitors used as monotherapy or in combination with ICI represent a promising therapeutic option in targeting the TGF-β pathway.

Another class of TGF- β inhibitors are ligand traps, which prevent the binding of TGF-β to its receptors. AVID200 is a ligand trap currently being studied in patients with advanced solid tumors in a phase I clinical trial (Table 1, NCT03834662). In addition to the aforementioned therapies that inhibit the TGF-β signaling pathway, bifunctional fusion proteins that bind to and block both TGF-β as well as CTLA-4 or PD-L1 have also been developed and are currently undergoing early-phase clinical trials (Table 1) [100]. These bifunctional fusion proteins, such as bintrafusp alfa (M7824), are based on TβRII ligand trap and act on TGF-β1 and TGF-β3. An anti-CTLA-4-TβRII studied in a mouse model of breast cancer demonstrated superior activity against tumors compared to single-agent or combination TGF-β antibody and an anti-CTLA-4 antibody [100]. Bintrafusp alfa has been studied in the phase I setting in several malignancies, including platinum-refractory NSCLC. In a phase I study evaluating bintrafusp alfa at 500 mg every 2 weeks versus the recommended phase 2 dose of 1200 mg every 2 weeks, the overall response rate (ORR) was 21.3% (17 out of 80 patients) [101]. Among patients on a 1200 mg dose, patients whose tumors had 80% or greater PD-L1 expression showed an ORR of 85.7% (6 out of 7 patients). There are currently over 20 ongoing trials involving bifunctional fusion proteins in advanced malignancies.

**Table 1:** Completed clinical trials involving anti-TGF-β pathway therapies with or without ICI in the setting of advanced/metastatic UC or advanced solid tumors.

Setting	Anti-TGF-β Pathway Agent	Mechanism of Action of Anti-TGF-β Agent	ICI	Results	Citation
Phase I in patients with metastatic melanoma and metastatic renal cell carcinoma	Fresolimumab	Monoclonal antibody	N/A	1 partial response (1/29 = 3.4%), 6 stable disease (6/29 = 20.7%). Median PFS of 24 weeks	Morris JC, et al. PloS One. 2014;9(3):e90353. [91]
Phase I in patients with glioma	Galunisertib	Small-molecule inhibitor	N/A	Clinical response (complete response + partial response + stable disease) in 12/56 (21.4%) patients	Rodon J, et al. Clin Cancer Res Off J Am Assoc Cancer Res. 2015 Feb 1;21(3):553–60. [98]
Phase Ib study in patients with unresectable HCC	Galunisertib	Small-molecule inhibitor	N/A	1 partial response (1/14 = 7.1%), 11 stable disease (11/14 = 78.6%)	Ikeda M, et al. Invest New Drugs. 2019 Feb;37(1):118–26. [99]
Phase I in patients with several malignancies, including platinum-refractory NSCLC	Bintrafusp alfa	Bifunctional fusion protein	N/A	Overall response rate was 21.3% (17 out of 80 patients).	Paz-Ares L, et al. J Thorac Oncol Off Publ Int Assoc Study Lung Cancer. 2020 Jul;15(7):1210–22. [101]

**Table 2:** Ongoing (highlighted in bold) and future clinical trials involving anti-TGF-β pathway therapies with or without ICI in the setting of advanced/metastatic UC or advanced solid tumors.

Setting	Anti-TGF-β Pathway Agent	Mechanism of Action of Anti-TGF-β Agent	ICI	ClinicalTrials.gov Identifier	Status
Phase I study in patients with advanced solid tumors	SAR439459	Pan-neutralizing anti-TGF-β antibody	With or without cemiplimab	NCT03192345	Recruiting
Phase Ib study in advanced/unresectable solid tumors (TACTIC Trial)	SAR439459	Pan-neutralizing anti-TGF-β antibody	Cemiplimab	NCT04729725	Recruiting
Phase I study in advanced malignancies	NIS793	Monoclonal antibody	PDR001	NCT02947165	Active, not recruiting
**Phase II, open-label study in mUC failing ICI**	**Vactosertib**	**Oral inhibitor**	**Durvalumab**	**NCT04064190**	**Not yet recruiting**
**Phase II study in ICI naïve and refractory mUC**	**Bintrafusp alfa (M7824)**	**Bifunctional fusion protein**	**N/A**	**NCT04501094**	**Recruiting**
Phase I study in adult patients with locally advanced or metastatic tumors	SRK-181	Monoclonal antibody	Approved anti-PD-L1 therapy for each tumor type	NCT04291079	Recruiting
Patients with advanced cancer	LY3200882	Small-molecule inhibitor	Pembrolizumab	NCT04158700	Withdrawn

Other therapies targeting the TGF-β signaling pathway include antisense oligonucleotides (ASOs). ASOs are synthetically engineered single-stranded oligodeoxynucleotides that can alter RNA and ultimately affect protein expression [102]. AP12009 is one such ASO that targets TGF-β2 and has been studied in patients with glioma with demonstrated clinical benefit, and is currently being studied in several other tumor types [103]. Other ASOs that are currently studied in the pre-clinical trial setting in tumors such as non-small-cell lung cancer, prostate adenocarcinoma, and colorectal cancer include AP11014 and AP15012 [104].

## 6. Conclusions

In summary, the TGF-β signaling pathway may represent a resistance mechanism to immune checkpoint inhibitors (ICI) in mUC through its interaction with EMT and its effects on the TME. Several pre-clinical and clinical studies combining ICI with novel inhibitors of the TGF-β pathway across multiple solid tumors including mUC have demonstrated a promising treatment response in early-phase clinical trials. Given that the majority of patients with mUC (~80%) do not respond to ICI monotherapy, further studies evaluating ICI in combination with TGF-β pathway inhibition are urgently warranted to improve the overall clinical benefit.
